# Motion, fixation probability and the choice of an evolutionary process

**DOI:** 10.1371/journal.pcbi.1007238

**Published:** 2019-08-05

**Authors:** Francisco Herrerías-Azcué, Vicente Pérez-Muñuzuri, Tobias Galla

**Affiliations:** 1 Theoretical Physics, School of Physics and Astronomy, The University of Manchester, Manchester, United Kingdom; 2 Group of Nonlinear Physics, Faculty of Physics, University of Santiago de Compostela, Santiago de Compostela, Spain; University of California Irvine, UNITED STATES

## Abstract

Seemingly minor details of mathematical and computational models of evolution are known to change the effect of population structure on the outcome of evolutionary processes. For example, birth-death dynamics often result in amplification of selection, while death-birth processes have been associated with suppression. In many biological populations the interaction structure is not static. Instead, members of the population are in motion and can interact with different individuals at different times. In this work we study populations embedded in a flowing medium; the interaction network is then time dependent. We use computer simulations to investigate how this dynamic structure affects the success of invading mutants, and compare these effects for different coupled birth and death processes. Specifically, we show how the speed of the motion impacts the fixation probability of an invading mutant. Flows of different speeds interpolate between evolutionary dynamics on fixed heterogeneous graphs and well-stirred populations; this allows us to systematically compare against known results for static structured populations. We find that motion has an active role in amplifying or suppressing selection by fragmenting and reconnecting the interaction graph. While increasing flow speeds suppress selection for most evolutionary models, we identify characteristic responses to flow for the different update rules we test. In particular we find that selection can be maximally enhanced or suppressed at intermediate flow speeds.

## Author summary

Whether a mutation spreads in a population or not is one of the most important questions in biology. The evolution of cancer and antibiotic resistance, for example, are mediated by invading mutants. Recent work has shown that population structure can have important consequences for the outcome of evolution. For instance, a mutant can have a higher or a lower chance of invasion than in unstructured populations. These effects can depend on seemingly minor details of the evolutionary model, such as the order of birth and death events. Many biological populations are in motion, for example due to external stirring. Experimentally this is known to be important; the performance of mutants in E. coli populations, for example, depends on the rate of mixing. Here, we focus on simulations of populations in a flowing medium, and compare the success of a mutant for different flow speeds. We contrast different evolutionary models, and identify what features of the evolutionary model affect mutant success for different speeds of the flow. We find that the chance of mutant invasion can be at its highest (or lowest) at intermediate flow speeds, depending on the order in which birth and death events occur in the evolutionary process.

## Introduction

The study of the success of mutants in evolving populations is a well-established focus area of research in computational biology. Approaches to this problem range from mostly theoretical work to direct application to specific biological systems [1–26]. The simplest way of modelling evolution is to dispense entirely with the notion of space and population structure, and to assume that the only relevant factors are the frequencies of the different types of individuals in the population [27–29]. Each individual in such an unstructured population can interact with all other individuals at all times.

If individuals are distributed in space, and have a limited range of interaction, the population becomes structured. Not every individual can interact with every other individual at all times. It is then helpful to consider the interaction graph of the population [25, 30–34]. Nodes of these networks represent individuals, and links between nodes stand for potential interactions. Evolutionary events take place between pairs of individuals connected by a link. The case of an unstructured population is recovered if links exist between any two individuals at all times; the interaction graph is then said to be complete. Evolution on simple graphs has been characterised mathematically (see for instance Refs. [5–8, 30–32] and references therein), and theoretical modelling has been linked to biological systems, see e.g. [24–26].

One main conclusion of this line of work is that population structure has the potential to change the dynamics of evolutionary processes [25–27, 32–45]. For example, species that would be selected against in an unstructured population are found to organise in clusters on networks, and in this way they can coexist with fitter types, or even eradicate a resident species.

In many stylised models of evolution birth and death events are coupled: when one individual dies, another one is born. This maintains a constant overall population size and facilitates the mathematical analysis [27–29]. It is recognised that the sizes of real populations fluctuate. Nevertheless, models with constant population size carry biological relevance; for example, they have been used to describe cancer cell populations [9–15], competition in microbial systems [16–23] and the evolution of cooperation [46–48]. Analytical results are available for models with constant population size, and serve as important benchmark; the effects of gradually introducing additional features can be tested against this baseline.

In this context it has been shown that certain interaction graphs can promote selection, while others suppress it [24–26, 36–43]. One key quantity used to characterise selection is the probability with which an invading mutant reaches fixation in an otherwise wildtype population. Specifically, selection is said to be ‘amplified’ or ‘suppressed’ when the fixation probability of a fitter mutant is higher or lower than in unstructured populations, respectively. The availability of analytical results for stylised models is particularly convenient for making such comparisons.

The effects of a given population structure on the success of an invading mutant can depend on the microscopic process chosen to model evolution. For example, the order of birth and death events can reverse the effect of the population structure [41, 43, 49]. One can further distinguish between models with global and with local selection [1, 2, 27], and again the outcome of evolution can be different depending on what type of model is used. Choosing the right evolutionary model for a given biological system is therefore an important and intricate task.

Further complications arise if the members of the populations are in motion. The interaction graph then becomes dynamic, making mathematical approaches more difficult. At the same time motion is a ubiquitous feature of biological systems, present for example due to self-propulsion of microswimmers by means of flagella [50], or advection of bacteria in a fluid environment [51]. The movement of the population has been found to modify the performance of a mutant. For example, differences in fixation probabilities have been found in static versus stirred populations of *Escherichia coli* [52–54].

Recognising that motion is an important aspect of evolutionary systems, the purpose of our study is to investigate how the success of an invading mutant is affected by dynamic population structure. In order to be able to compare our results with those in unstructured populations and in populations with static interaction networks, we restrict the analysis to the most common models used in this context. Specifically, we focus on birth-death and death-birth processes in populations of constant size.

A common way of implementing motion in models of evolution is migration; in these models individuals move to neighbouring sites on the interaction graph [24, 55–60]. Alternatively, adaptive networks have been considered; in these networks individuals can re-wire their links to other members of the population, usually with preference for links between individuals of similar types [34, 61–65]. A separate approach assumes that organisms move randomly [66, 67]. Work based on passive motion in flows includes [68]; the evolution of movement in algae in water columns has been studied in [69]. Much of this existing work on evolution in systems with mobility is based on deterministic models, continuous both in space and time; see however [70] for a stochastic approach. Work using continuous deterministic or stochastic reaction-diffusion-advection equations to describe populations of mobile individuals can be found in [71–73].

In this paper, we study populations that are not self-propelled and use the type of motion one could expect in dynamic gaseous or aqueous environments. Specifically, the motion is due to a flow of the medium in which the population resides. In other words, it is the type of motion one would expect when populations are stirred or mixed by external forces. The movement is not constrained by the current interaction network, and the interaction graph itself is dynamic. Similar implementation of motion can be found in refs. [60, 71–79]. Using this type of motion, and systematically studying its effects at different flow speeds, allows us to connect known results for static heterogeneous graphs and for well-stirred populations.

We focus on the rate of successful fixation of a single invading mutant in populations of discrete individuals. We find that the way in which the flow affects its success depends on the choice of the evolutionary update rules. Specifically we find differences between birth-death dynamics and death-birth processes, and our study shows that it is important to consider whether selection is global or local in the evolutionary model. We note that the distinction between death-birth versus birth-death dynamics is not usually possible in models based on continuous population densities.

We identify three main factors contributing to the effects flow has on the evolution of mutants in discrete populations: how well connected the initial mutant is with the rest of the population, the opportunities mutants have to organise in clustered groups, and how long individuals remain connected for as the flow moves them in space. These factors are influenced by the speed of the flow and, depending on the evolutionary update rule, they can amplify or suppress selection relative to unstructured populations. We speculate that this may be used to discriminate between different stylised models. In some experimental settings flow can be controlled externally, or situations without flow can be compared to those with fast flows. If such data is available, systematically studying the behaviour of different computational models of evolution in flowing populations can help to select the update mechanism which best captures the features of the biological system at hand.

## Methods

We use the same setup as ref. [79], and consider a population of fixed size *N* composed of individuals of two species (wildtype and mutant). Unless specified otherwise, we use *N* = 100. Individuals take positions in space within the two-dimensional domain 0 ≤ *x*, *y* < 1 with periodic boundary conditions. Particles are subject to a continuous-time flow, moving them around in space, and to evolutionary dynamics, which change the frequencies of the two species in the population.

The motion of the particles is simulated through the so-called parallel shear flow [80, 81]; we discuss the validity of our results for different flow fields in the Discussion section. The velocity field of this flow is periodic in time, except for a random phase described below. During the first half of each period particles are moved vertically; the speed of each individual depends on the horizontal component of their position. During the second half of the period individuals move horizontally, with speeds dependent on their vertical positions. We write *v*_*x*_(*x*, *y*, *t*) and *v*_*y*_(*x*, *y*, *t*) for the velocity components of a particle at position (*x*, *y*) at time *t*. Specifically, we use

$$\begin{array}{cccc} v_{y}\left(x,y,t\right)=0,\; & \;v_{x}\left(x,y,t\right)=V_{\text{max}}\;\text{sin}[2\pi y+\psi ], & & \;\text{for}\;t\in [nT,nT+T/2),\\ v_{x}\left(x,y,t\right)=0,\; & \;v_{y}\left(x,y,t\right)=V_{\text{max}}\;\text{sin}[2\pi x+\psi ], & & \;\text{for}\;t\in [nT+T/2,(n+1)T), \end{array}$$

with *n* = 0, 1, 2, …. The constant *V*_max_ sets the amplitude of the flow, and *T* the period. The phase *ψ* is drawn randomly from the interval [0, 2*π*) at the beginning of each half-period. Due to this random phase, the flow mimics chaotic motion; the trajectories of individuals who are initially close to each other diverge over time. At long times, the distribution of individuals moved by this flow is uniform in space [80, 81].

The evolutionary process is implemented through coupled birth and death events. The order in which reproduction and removal take place is important, and so we will distinguish between birth-death and death-birth processes. The evolutionary dynamics occur on an undirected interaction graph, dynamically generated by the flow. Specifically, we will say that one individual is a neighbour of another if they are within a distance *R* of each other. In the main text we focus on a scenario in which mutation is separate from reproduction, and therefore we initialize our simulations with a single initial mutant chosen uniformly at random. The case of mutation during a reproduction event is more adequately simulated by temperature initialization, which we discuss in part D of S1 Text in the Supporting Information.

Individuals are in continuous motion, but evolutionary events occur at discrete times, *t* = Δ*t*, 2Δ*t*, … in our model. Simulations are then implemented as follows:

1. At *t* = 0, a population of *N* particles is placed into the spatial domain at designated initial positions. These define an initial interaction graph. Of these individuals, *N* − 1 are wildtype and one is a mutant.
2. The individuals are moved by the flow for a time interval Δ*t*, leading to a new interaction graph.
3. An individual is chosen from the entire population. In the case of a birth-death process, it is designated to reproduce; for death-birth processes it is designated to die.
4. One of the neighbours of this individual is chosen to be replaced (birth-death) or to reproduce (death-birth).
5. The individual chosen for death adopts the species (wildtype or mutant) of the reproducing individual.
6. Repeat from step 2.

Due to the coupled birth and death events the size of the population in the model is constant over time. This allows for comparison against analytical results for populations on complete graphs as discussed below. It also facilitates the memory allocation required for the simulations; the individuals’ locations, species type and the adjacency matrix of the population are arrays of fixed size.

In each evolutionary step two individuals are chosen. For simplicity, we will say that an individual is ‘*picked*’ when it is chosen at random, disregarding fitness differences, or that it is ‘*selected*’ when the choice of the individuals is made through competition, i.e., proportional to fitness. For the latter case we focus on frequency-independent selection; we set the wildtype fitness to one, and write *r* for the fitness of the mutant species. Consider for example a group of *n*_*w*_ wildtype individuals and *n*_*m*_ mutants. A mutant would be selected to reproduce from this group with probability *rn*_*m*_/(*rn*_*m*_ + *n*_*w*_), or a wildtype with probability *n*_*w*_/(*rn*_*m*_ + *n*_*w*_). If selection is for death we proceed similarly, but with *r* replaced by 1/*r*. In this way, mutants are less likely to die than wildypes if *r* > 1. For *r* < 1 the mutant species is selected against. The simulation results shown in this paper focus on advantageous mutants; we set *r* = 1.05 throughout.

Selection proportional to fitness can take place either in step 3 of the above algorithm (when an individual is chosen from the entire population) or in step 4 (when it is chosen from the neighbours of an individual). We refer to these cases as *global* and *local* selection, respectively. Since we distinguish between birth-death and death-birth processes, four combinations are possible: global birth-death (**Bd**), global death-birth (**Db**), local birth-death (**bD**) and local death-birth (**dB**). The capital letter in these acronyms indicates that selection dependent on fitness occurs in the respective step. In principle, one could also consider processes in which individuals are chosen proportional to fitness in both steps of the algorithm (**BD**, **DB**) [1, 2]. In order to be able to disentangle the effects that the flow has on fixation probabilities due to local or global selection, we limit the discussion in the main text to scenarios in which selection acts either globally or locally, but not both. The **BD** and **DB** processes are discussed in part C of S1 Text in the Supporting Information.

**Fig 1:**
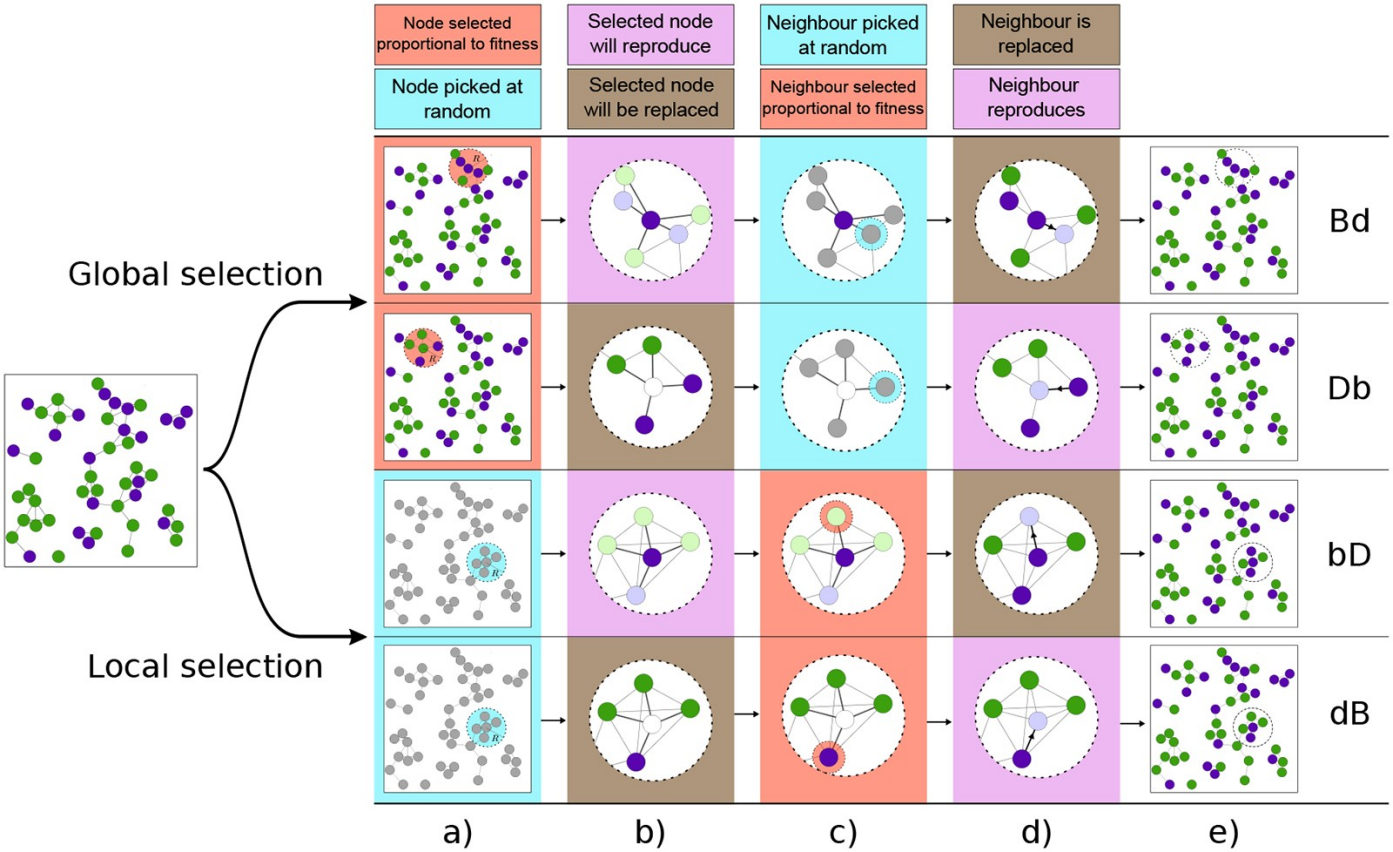
Illustration of the update rules. Each row represents one of the different evolutionary update mechanisms. The columns indicate the different steps of each evolutionary event. In column a) an individual is chosen from the whole population; it can be *‘selected’* through competition by fitness (red shading), or *‘picked’* at random, irrespective of its species (blue shading). This node is destined to either reproduce (pink shading), or to be replaced (brown shading), as shown in column b). Column c) indicates that one neighbour of this node is either *selected* (red), or *picked* (blue). This second node is destined to reproduce (pink), or to be replaced (brown), shown in column d). Column e) shows the result of the evolutionary event; the node chosen to reproduce places an offspring in place of the node chosen to die. Each row is composed of one box of each colour; the sequence of the colours distinguishes the different processes. From top to bottom, the rows correspond to: (i) global birth-death process (**Bd**): an individual is *selected* from the whole population to reproduce, and one of its neighbours is *picked* to be replaced by the first individual’s offspring; (ii) global death-birth process (**Db**): an individual is *selected* to die from the whole population, and one of its neighbours is *picked* to place an offspring in its place; (iii) local birth-death process (**bD**): an individual is *picked* from the whole population to reproduce, and one of its neighbours is *selected* to die; (iv) local death-birth process (**dB**): an individual is *picked* from the whole population to die, and one of its neighbours is *selected* to reproduce.

We illustrate the different evolutionary processes in Fig 1. The upper two rows correspond to processes in which competition takes place among the entire population (*global selection*). In the lower two rows the first node is picked irrespective of fitness, and competition takes place only among the neighbours of this node (*local selection*). A step-by-step description of each of the processes can be found in the figure caption.

One important characteristic of the flow is the typical timescale over which the set of neighbours of a given individual is renewed. More precisely, the set of neighbours of a given individual at time *t*, and at a later time *t* + *τ*, will be uncorrelated provided *τ* is sufficiently large (see ref. [79], and part A of S1 Text in the Supporting Information). This renewal time is in turn determined by the parameters *V*_max_, *T* and *R*; following refs. [82, 83], we use *V*_max_ = 1.4 and *T* = 1 throughout, and choose an interaction radius of *R* = 0.11.

This choice of parameters leads to an estimate for the network renewal time of *τ* ≈ 6.4 (see part A of S1 Text in the Supporting Information for details). That is, the set of neighbours of one individual at one time is uncorrelated from its set of neighbours approximately six and a half flow periods earlier. It remains to specify how frequent evolutionary events are, i.e. to define the time step Δ*t* in the simulation described above. We treat this as a model parameter, and use *S* = *N*Δ*t*/*T* to quantify the number of generations elapsed in one flow period. Thus, *S* indicates the speed of the flow relative to that of evolution. For small *S*, individuals move relatively little between evolutionary events (‘slow flow’). Large values of *S* describe fast flows. From here on, we will refer to *S* as the *speed* of the flow, and investigate the outcome of evolution for different choices of this parameter. The flow speed *S* is understood throughout as relative to the rate of evolutionary events. We note that the inverse of *S* is related to the Damköhler number in fluid dynamics [84–86].

## Results

### Effects of the flow speed on the fixation probability

We first address the case in which the initial coordinates of each individual are drawn from a uniform distribution on the domain 0 ≤ *x*, *y* < 1. The initial interaction graph is then a random geometric graph (RGG) [87].

For any non-zero flow rate (*S* > 0) any member of the population can eventually interact with any other individual, even if they were not connected on the initial interaction graph. This is due to the mixing properties of the flow, and means that no individual can indefinitely remain isolated from the rest of the population. As a consequence, the final outcome of the evolutionary process is either fixation or extinction of the mutant.

**Fig 2:**
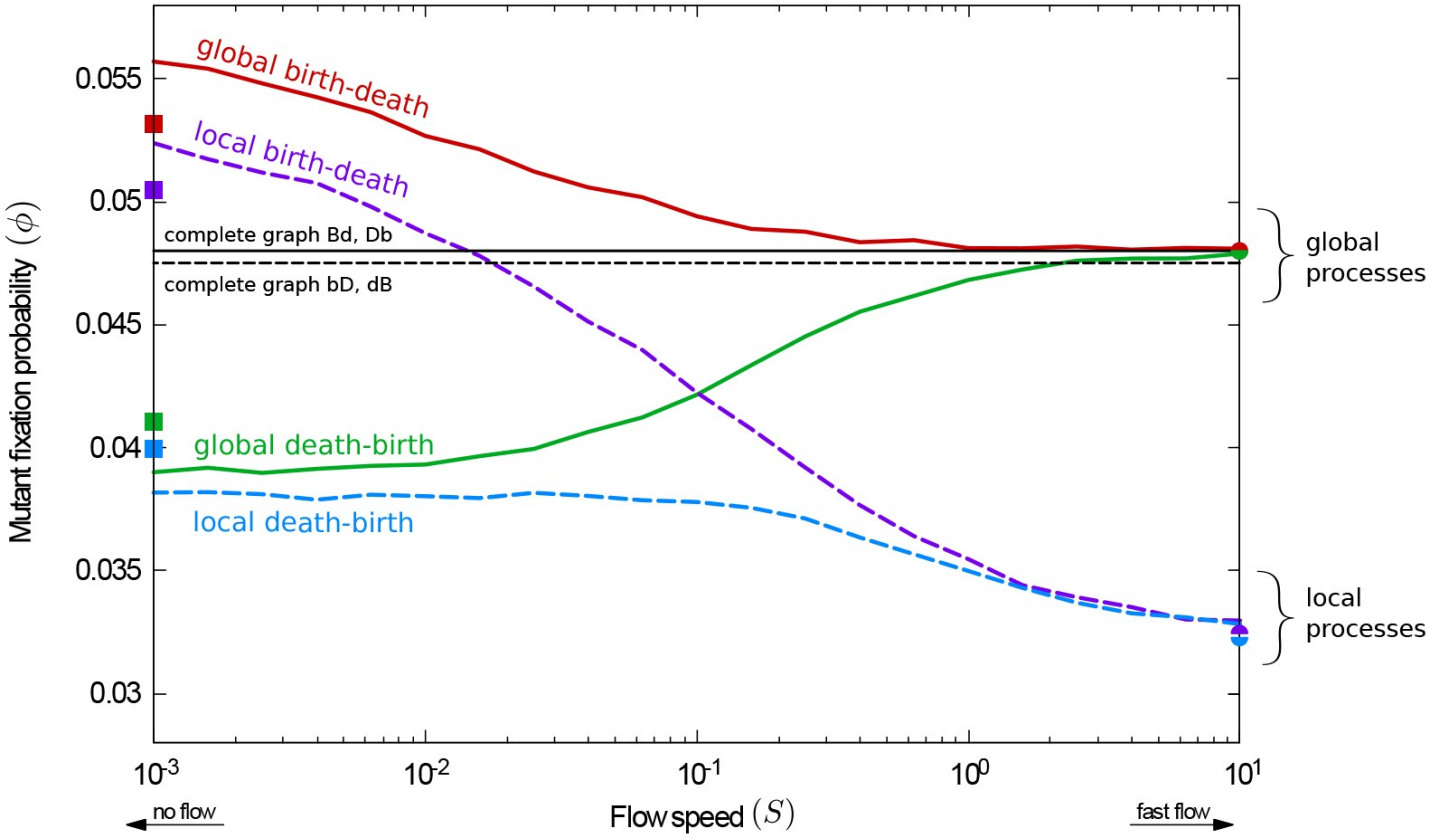
Fixation probability as a function of the flow speed for unrestricted random initial positions (random geometric graphs, RGGs). For the global death-birth process, increasing the flow speed increases the fixation probability. The reverse is found for the remaining three processes. Circle markers show fixation probabilities in the fast-flow limit; square markers are results for fixed connected random geometric graphs (CRGGs); see text for further details. The fixation probabilities on a complete graph are shown for reference.

The fixation probability, *ϕ*, for a beneficial mutation is depicted in Fig 2 as a function of the flow speed, *S*. We show simulation results for the four different evolutionary processes **bD**, **dB**, **Bd**, and **Db**. Each data point is obtained from an ensemble of realisations. For comparison, we also show the fixation probability on a complete graph, *ϕ*_CG_. By definition, *ϕ*_CG_ is independent of the flow speed, as all individuals interact with all others at all times. On complete graphs the fixation probability for global and local selection processes differ by a small amount [1].

Several interesting features can be observed in Fig 2: For slow flows, the order of reproduction and removal is found to have a strong effect on the fixation probability, and it is less relevant whether selection takes place in the first or the second step of each evolutionary event. For the local and global death-birth processes (**dB**, **Db**) the fixation probability is lower than on a complete graph, as shown by the green and blue lines in Fig 2. Conversely, both **Bd** and **bD** show a higher fixation probability than on complete graphs (red and purple lines).

In the limit of fast flows, however, the outcome of evolution is mostly determined by whether selection is global or local, and not by the order of the reproduction and removal events (birth-death vs. death-birth). Specifically, when selection acts locally the fixation probability of the mutant is lower than on a complete graph (purple and blue lines). In contrast, when selection is global the fixation probability is the same as on a complete graph (red and green lines).

These observations indicate unique responses of the fixation probability to the flow speed for the different processes. For the **Db** process (continuous green line in Fig 2) the mutant’s probability of success increases with the speed of the flow. For the **Bd** process (continuous red line), the fixation probability decreases with increased flow speed, but is always greater than or equal to the one on a complete graph, *ϕ* ≥ *ϕ*_C*G*_. In contrast, the fixation probability for a **dB** process (dashed blue line) is always smaller than *ϕ*_C*G*_. Finally, for the **bD** process (dashed purple line) the fixation probability is higher than on a complete graph when the flow is very slow, but decreases at higher flow speeds and eventually becomes lower than on the complete graph. The **bD** process is the only case in which we observe a transition from amplification to suppression of selection (relative to the complete graph) as the flow speed is increased.

In order to gain some insight into these observations, we first describe the dynamics in the limit of fast flows, summarising the results of ref. [79]. Then we discuss the no-flow limit, and subsequently the transition between the two extremes, at intermediate flow speeds.

#### Fast-flow limit: Evolution of well-stirred populations

When the flow is sufficiently fast the probability that any two particles are neighbours at the time of an evolutionary event is the same, irrespective of whether they were neighbours at the previous event or not [79]. In global processes selection takes place when the first individual is chosen, i.e., competition acts amongst the whole population. Then, a second individual is picked at random from the neighbours of this first individual. Since any individual is equally likely to be neighbours of the individual selected in the initial step, the second, random pick, is equivalent to a random pick from the entire population. Therefore, in the limit of fast flows the fixation probability of global processes coincides with the one on complete graphs, as observed in Fig 2.

For local processes, on the other hand, in each evolutionary event the first individual is chosen at random from the entire population, irrespective of fitness. Competition then takes place between the neighbours of this individual. Although all members of the population are equally likely to be part of this neighbourhood, at any one time the group of neighbours is a random subset of the population. This subset may not reflect the composition of the population as a whole, which can be shown to lead to suppression of selection [79]. We briefly illustrate this for the case of a very small interaction radius; the majority of individuals then have at most one neighbour at any given time. Since this neighbour is the only contestant in local selection, fitness is irrelevant. Therefore, as the interaction radius becomes small the fixation probability of the mutant approaches the limit of neutral selection. When the interaction radius is large, however, it is more likely that the group of neighbours is large as well, and that population-wide frequencies are accurately represented. Therefore, the suppression effect relative to the complete graph is reduced. If the interaction range is so large that all individuals are connected with all other individuals at all times, a complete interaction graph is recovered.

Analytical results can be obtained for all four processes in the limit of very fast flows [79]. Predictions from this theoretical approach are shown as filled circles on the right edge of Fig 2.

#### No-flow limit: Evolution on static heterogeneous graphs

On the left-hand side of Fig 2 the flow is so slow that the evolutionary dynamics effectively take place on fixed graphs. Evolutionary processes on static graphs have been widely discussed in the literature (see e.g. [25, 31, 42] and references therein). The focus is often on characterizing specific graphs or graph structures, which can amplify or suppress selection [88–90]. Notably, the authors of ref. [41] report that most undirected graphs amplify selection for birth-death processes, but suppress selection for death-birth processes. However, these findings are only given for relatively small networks, and only for processes in which selection acts in the reproduction step (**Bd** and **dB**).

**Fig 3:**
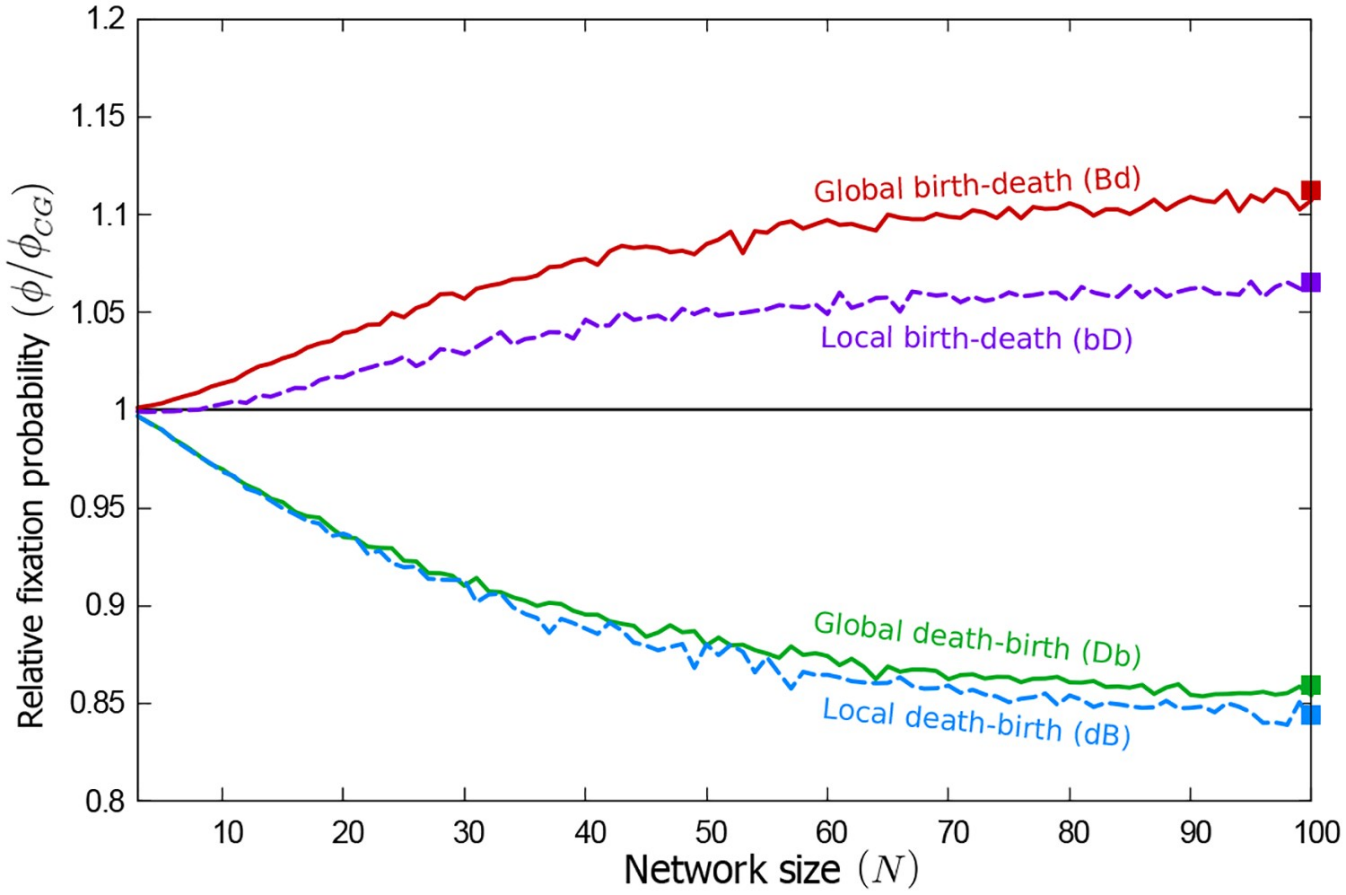
Fixed heterogeneous graphs amplify selection for birth-death processes and suppress it for death-birth processes. The figure shows the fixation probability of an invading mutant (*ϕ*), averaged over static CRGGs. Data is shown relative to the corresponding fixation probability on a complete graph (*ϕ*_*CG*_). Regardless of the population size, selection is amplified for **Bd** and **bD** processes, and suppressed for **Db** and **dB** processes.

In order to obtain a more complete picture, we measured in simulations the fixation probability of a single mutant on networks of different sizes, averaged over different static heterogeneous graphs. Each graph is generated by placing individuals at random in the spatial domain (see Methods), resulting in a random geometric interaction graph. It is possible that a graph generated in this way consists of several disconnected components. In the absence of flow, the mutant then cannot reach fixation. We therefore restrict simulations to graphs with a single connected component and henceforth use the term *connected* random geometric graphs (CRGGs). We present results for the different evolutionary processes as a function of the size of the graph in Fig 3.

The data shows that the average fixation probability of a single mutant on CRGGs is higher than on the complete graph for birth-death processes, *ϕ* ≥ *ϕ*_C*G*_. For death-birth processes, on the other hand, *ϕ* ≤ *ϕ*_C*G*_. This is in line with the results reported in ref. [41] for small graphs. The data in Fig 3 confirms that the amplification of selection (for birth-death processes) or suppression (for death-birth processes) is present regardless of the size of the network, if an average over many graphs is taken. The rightmost data points in Fig 3 correspond to a population of the same size as the one in Fig 2.

Intuition regarding the amplification or suppression of selection on static networks can be gained by studying the connectivity of the initial mutant (see refs. [43, 49, 91–93]). For death-birth processes, these studies find that the success of an advantageous mutant increases with its degree; for birth-death processes, its success decreases with connectivity. This can be understood in the following way: In each evolutionary event two individuals are chosen, the first from the entire population, and the second as a neighbour of the first. The degree of an individual does not affect its chances of being chosen in the first step, irrespective of whether selection acts in this step or not. However, the probability of being neighbours with the initial individual is higher for well connected individuals than for individuals with a low degree. Under birth-death processes, higher connectivity of the mutant therefore results in a higher chance of being replaced. For death-birth processes it results in a higher chance of reproduction.

**Fig 4:**
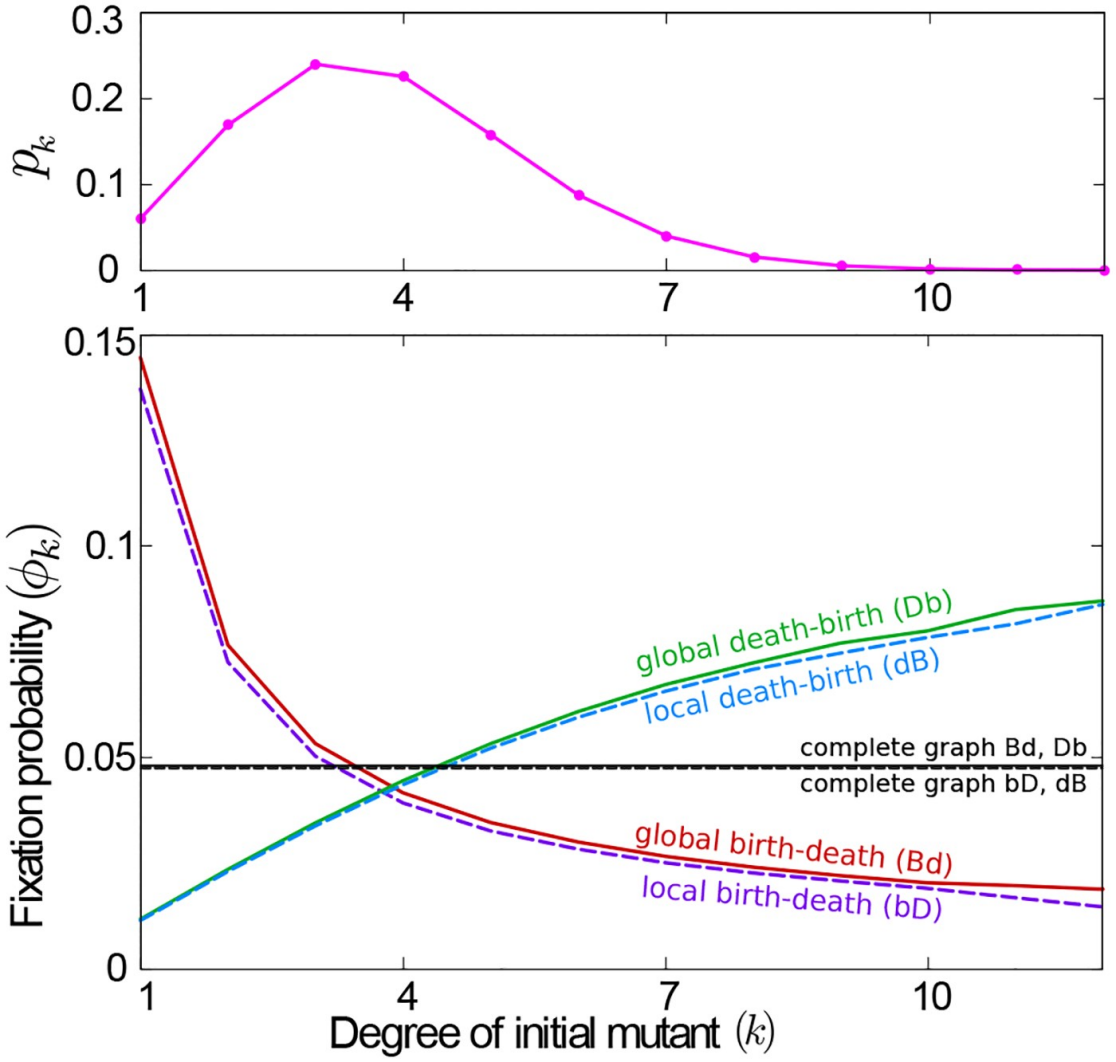
Significance of the degree of the initial mutant. The upper panel shows the degree distribution, *p*_*k*_, of the ensemble of connected random geometric graphs (CRGGs), obtained by placing *N* = 100 individuals into the spatial domain 0 ≤ *x*, *y* ≤ 1 with uniform distribution, and using an interaction radius *R* = 0.11 and periodic boundary conditions. The lower panel shows the fixation probability obtained from simulating the evolutionary process on these graphs, as a function of the degree of the initial mutant. For the two death-birth processes the mutant’s success is below the one on a complete graph if its degree is low, and above *ϕ*_*CG*_ at high connectivity. The reverse is found for the two birth-death processes. Data points have been connected as a visual guide.

In the literature these predictions have been tested for **Bd** and **dB** processes [43, 49, 91–93]. In the lower panel of Fig 4 we verify that the argument extends to all four evolutionary update rules defined above. We show, for CRGGs, the fixation probability of a mutant, *ϕ*_*k*_, as a function of its degree, *k*. For the global and local death-birth processes (**Db**, **dB**) the mutant’s success is lower than on a complete graph when the mutant is sparsely connected, but larger if it is highly connected; the reverse is found for global and local birth-death processes (**Bd**, **bD**). These observations are consistent with the above reasoning.

In our model, the initial mutant is chosen uniformly at random from the members of the population. The probability that it has degree *k* is thus determined by the degree distribution of CRGGs. We write *p*_*k*_ for the probability of a random node to have degree *k* in such a graph, and show the degree distribution for networks of size *N* = 100 in the upper panel of Fig 4 for illustration. The overall probability of fixation of a single mutant is then *ϕ* = ∑_*k*_ *p*_*k*_*ϕ*_*k*_. Fixation probabilities obtained in this way are shown as square markers in Figs 2a and 3. The results reproduce the amplification and suppression of selection (for birth-death or death-birth processes, respectively) in the limit of slow flows. We attribute quantitative differences between the markers and lines in Fig 2 to effects of the non-zero flow and to the difference in initial conditions; the data shown as lines is obtained from simulations of slowly flowing populations in which the initial graph may consist of more than one component. In the following section we will discuss the difference due to initial positions in more detail.

#### Transition between fast-flow and no-flow limits

As seen above, the outcome of evolution in rapidly stirred populations is very different to that on static interaction graphs. With fast flows, local competition leads to suppression of selection; on the other hand, the success of a mutant is the same as on a complete graph if selection is global. When there is no flow, the order of the birth and death events in the evolutionary process is crucial. In this case, selection is amplified for birth-death processes and suppressed for death-birth processes. At intermediate flow speeds, a crossover between these two regimes is seen. We will now discuss this transition in more detail.

On fixed heterogeneous graphs, the degree of the initial mutant determines whether its chances of success are greater or smaller than on a complete graph. In the presence of flow the interaction network constantly changes, and the number of neighbours of any one individual thus varies over time. Classifying a member of the population as highly or poorly connected is then at best possible over limited time windows. If the flow is slow relative to evolution, many evolutionary events occur in such a time window, and the evolutionary dynamics can conclude before the degrees of nodes undergo significant changes. Therefore the amplification or suppression effect due to the degree of the mutant can still be observed. For faster flows, however, the interaction network changes so quickly that there is no clearly defined notion of a degree of an individual on the time scale of evolution. The amplification or suppression effect set by the initial heterogeneous network is then washed out.

At very fast flow speeds, the set of neighbours of the individual chosen in the first step of an evolutionary update effectively becomes a group drawn at random from the entire population. That is to say, the set of neighbours a given individual interacts with in one event will be uncorrelated from that in the previous evolutionary event involving this particular individual (see Methods). Therefore, suppression of selection sets in for local processes. The fixation probability of global processes, on the other hand, approaches the one on a complete graph, as described previously.

The main effects leading to the transition between the no-flow and the fast-flow limits are thus the increasing variability (over time) of the degree of individuals, and the increasingly random and uncorrelated composition of the groups of individuals taking part in evolutionary events. As a result of these two mechanisms, in Fig 2 we see a smooth transition between the two limits. The different responses of the fixation probability to the speed are a consequence of the limiting behaviours for very slow and very fast flows.

Although it is not immediately transparent from the results in Fig 2, the flow has further effects on the evolutionary process. For example, it removes the influence of the initial positions of the individuals in space. Another important feature, particularly at intermediate flow speeds, is that the evolutionary process takes place on slowly changing heterogeneous graphs. The dynamic network constantly splits into disconnected components, which later merge and form new components. This fragmentation promotes the formation of ‘clusters’—groups of nodes which are of the same species. This gives rise to further amplification or suppression effects, depending on the details of the evolutionary mechanics. In the following section, we explore these effects further.

### Effects of the initial positions of individuals

Our model describes a population in constant motion. It is then natural to assume that the positions of the individuals at the time the initial mutation occurs is drawn from the stationary distribution of the flow. For the periodic parallel shear flow this is the uniform distribution, used as an initial condition in the previous section. However, exploring different starting positions allows us to gain further insight into the effect of the flow on fixation probabilities.

### Connected random geometric graphs (CRGGs)

The data shown as lines in Fig 2 was obtained from simulations with random initial positions (RGGs) and non-vanishing flows. For this setup the interaction graph may not be connected, but fixation or extinction will still occur, provided there is non-zero flow. In order to explore the no-flow limit, in Figs 3a and 4 we focused on static heterogeneous graphs instead; studying fixation in the strict absence of flow only makes sense when the interaction graph consists of one single connected component, and so we restricted the discussion to connected random geometric graphs (CRGGs). As a result, comparison with the data in Fig 2 is difficult; in particular, we note the quantitative differences between the square markers, obtained from static connected graphs, and the limiting values of the data shown as lines in Fig 2, obtained from slowly moving populations started from RGGs.

**Fig 5:**
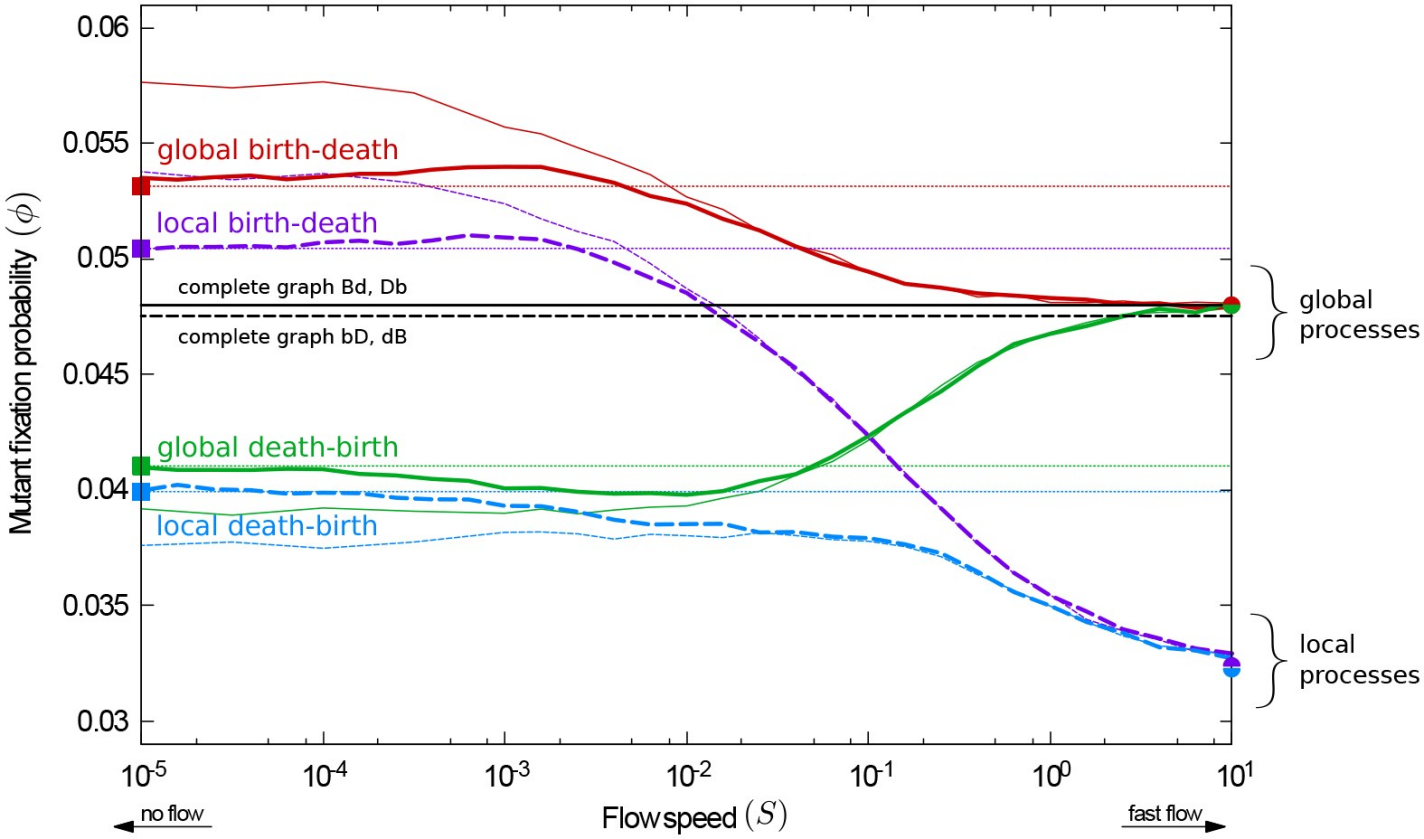
Comparison of fixation probability for simulations started from unrestricted and connected random geometric graphs (RGGs and CRGGs, respectively). The fixation probability as a function of the flow speed is shown as thick lines for simulations started on connected graphs; thin lines are for unrestricted initial positions (some of this data is also shown in Fig 2). Square markers indicate the fixation probabilities on *static* CRGGs; see text for details. The fixation probability on complete graphs is shown for reference. A minimum of *ϕ* is found for the **Db** process; maxima are discernible for **Bd** and **bD** when the dynamics are started from connected graphs. The effect of amplification/suppression of selection at slow flow speeds is more pronounced for simulations initialized from RGGs than from CRGGs.

For comparison, we show data obtained from mobile populations, but started on CRGGs, in Fig 5. The limiting values of the fixation probabilities for very slow flows (end of the tick lines on the left-hand side of the figure) now agree quantitatively with those obtained from static CRGGs (square markers).

The simulation data from Fig 2, from simulations with unrestricted random initial positions, is also shown in Fig 5 (thin lines). If the flow is sufficiently fast, initial conditions are immaterial. On the contrary, for slow flows the fixation probability, *ϕ*, for simulations started from unrestricted random graphs is different from that for connected initial conditions. For birth-death processes, *ϕ* is greater for the unrestricted case than for the connected one. The opposite is observed for death-birth processes. This indicates that the initial condition can have a significant effect on the outcome when then flow is slow.

**Fig 6:**
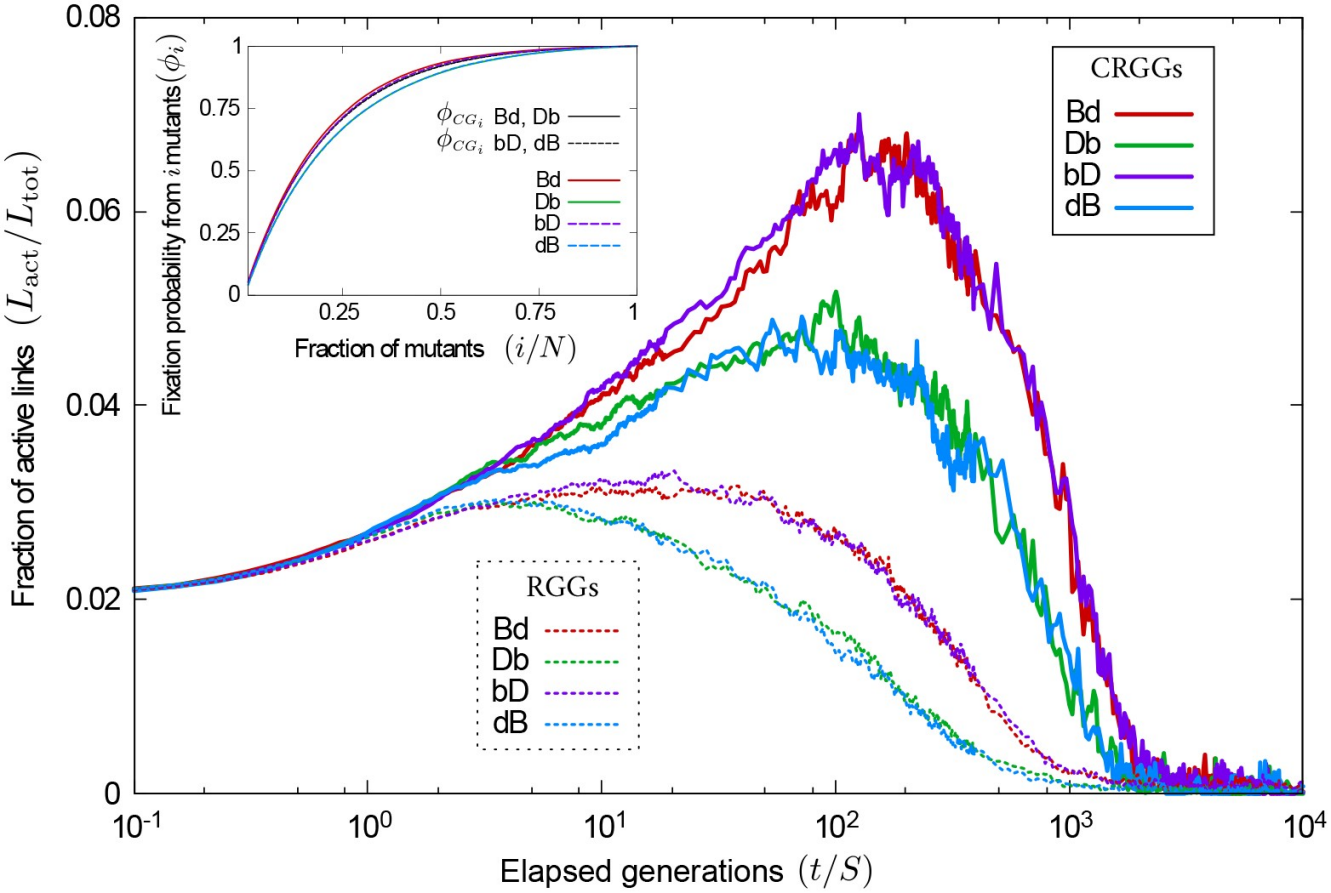
Fragmented initialization promotes the formation of clusters. The main panel shows the average proportion of active links as the evolutionary dynamics proceed. Thick lines correspond to simulations started from connected graphs (CRGGs); thin dotted lines to simulations initialized from unrestricted random positions (RGGs). The fraction of active links is lower for RGGs, regardless of the evolutionary process. **Inset**: Fixation probability of the mutant species, once there are *i* mutants in the population. When mutants are a minority, a small increase in their frequency greatly increases their fixation probability. Conversely, reducing their numbers when they are a majority has only minor effects on their chances of success. Simulations in the inset are initialized from CRGGs.

As briefly mentioned before, the fragmented nature of the unrestricted setup can isolate groups of nodes from the rest of the population. As the evolutionary dynamics proceed, this promotes the formation of clusters, i.e. parts of the graph in which all individuals are of the same species. Clusters arise from the spread of the mutation from individual mutants seeded in different parts of the system to their local neighbours. We note that this is not restricted to discrete individual-based models, but that similar domain formation can, in principle, be expected in models with continuous population densities and in continuous time. The degree of clustering can be quantified through the fraction of active links in the network, that is, the proportion of links between mutants and wildtypes among all links in the graph, *L*_act_/*L*_tot_. A small fraction of active links is an indicator of clustering. We show measurements of the fraction of active links in Fig 6 for both unrestricted and restricted random initial conditions (thin dotted lines and thick continuous lines, respectively). The data indicates that the fraction of active links is significantly larger when simulations are initialised on CRGGs than when started on RGGs.

The amplification or suppression of selection (for birth-death and death-birth processes, respectively) can then be supported by a similar argument to the one presented for the degree of the initial mutant. A smaller number of active links has the same effect as poor connectivity of the initial mutant; it does not affect the probability that the individual chosen in the first step of the evolutionary process is a mutant or a wildtype, but it reduces the probability that the individual chosen in the second step is of the opposite species (see also part B of of S1 Text in the Supporting Information).

In the early stages of the evolutionary process mutants are a minority, and are therefore less likely to be chosen in the initial step. A large number of active links then increases the chances that the neighbour of the initial individual is a mutant. Under birth-death processes this means that mutants are more likely to die; for death-birth processes they have more opportunities to reproduce. Therefore, a connected initial configuration (CRGGs), leading to a larger fraction of active links than arbitrary RGGs, reduces the fixation probability of a mutant under birth-death processes, and increases it for death-birth processes. This is in line with the results on the left-hand side of Fig 5; the fixation probabilities for RGGs (dotted lines) are higher than their counterparts on CRGGs for birth-death processes (red and purple lines), but lower for death-birth processes (green and blue lines).

This argument is only valid when mutants are less abundant than wildtypes. The effect is reversed at later stages of the evolutionary process (if mutants become a majority). However, the results presented in Fig 5 suggest that there is a net advantage for the mutant in having fewer active links, for birth-death processes, or in having increased inter-species connectivity, for death-birth mechanics. The inset in Fig 6 helps to understand this further. It shows the conditional fixation probability of the mutant species, given that a state with *i* mutants has been reached. The shape of the curves indicates that increasing the number of mutants in the population has stronger repercussions on the fixation probability when mutants are a minority (*i*/*N* ≤ 0.5) than when they are the majority (*i*/*N* ≥ 0.5). For death-birth processes, the selective effect due to increased active links drives the population composition to states with approximately equal frequencies of the two species. However, the mutants have more to gain (in terms of fixation probability) when their numbers are small than what they may lose when they are abundant. For birth-death processes, on the other hand, a large number active links acts in the opposite way; it hinders the spread of the mutant species when they are a minority and encourages it once they are abundant. Since more is lost in the early invasion than what can be gained at later stages, the overall fixation probability is lower than when there are fewer active links. The net effect of fragmentation (i.e., a reduced number of active links) is therefore amplification of selection for birth-death processes, and suppression for death-birth update rules.

The amplification/suppression effect caused by the fragmented nature of the network can also be noticed at intermediate flow speeds. In this regime, the flow is sufficiently fast to disrupt the initial network structure before the evolutionary process reaches its conclusion (fixation or extinction of the mutant); disconnected components then develop. At the same time the flow is also slow enough to allow the formation of organised clusters of mutants and wildtypes through the evolutionary dynamics. Indeed, for simulations started on connected graphs a minimum in the fixation probability as a function of the flow speed is discernible for the **Db** process (thick green line in Fig 5), and we also notice a shallow maximum for the **Bd** and **bD** processes (thick red and purple lines, respectively). The fragmentation from an initially connected network increases the fixation probability for birth-death processes and decreases it for death-birth processes. Movement of the population, and the resulting mixing between evolutionary events counteracts this amplification or suppression, driving fixation probabilities to their fast-flow limits. The balance of these two effects leads to the extrema in Fig 5.

### Square lattice

Regular lattices are particularly convenient for the study of fixation probabilities. The nodes are distributed equidistantly in space, and they all have the same number of neighbours. This means that analytical results can be obtained in the absence of flows. For example, the isothermal theorem [30] applies; the fixation probabilities of the global birth-death and death-processes are the same as those for complete graphs; only small deviations from *ϕ*_*CG*_ are expected for local-selection processes [1].

**Fig 7:**
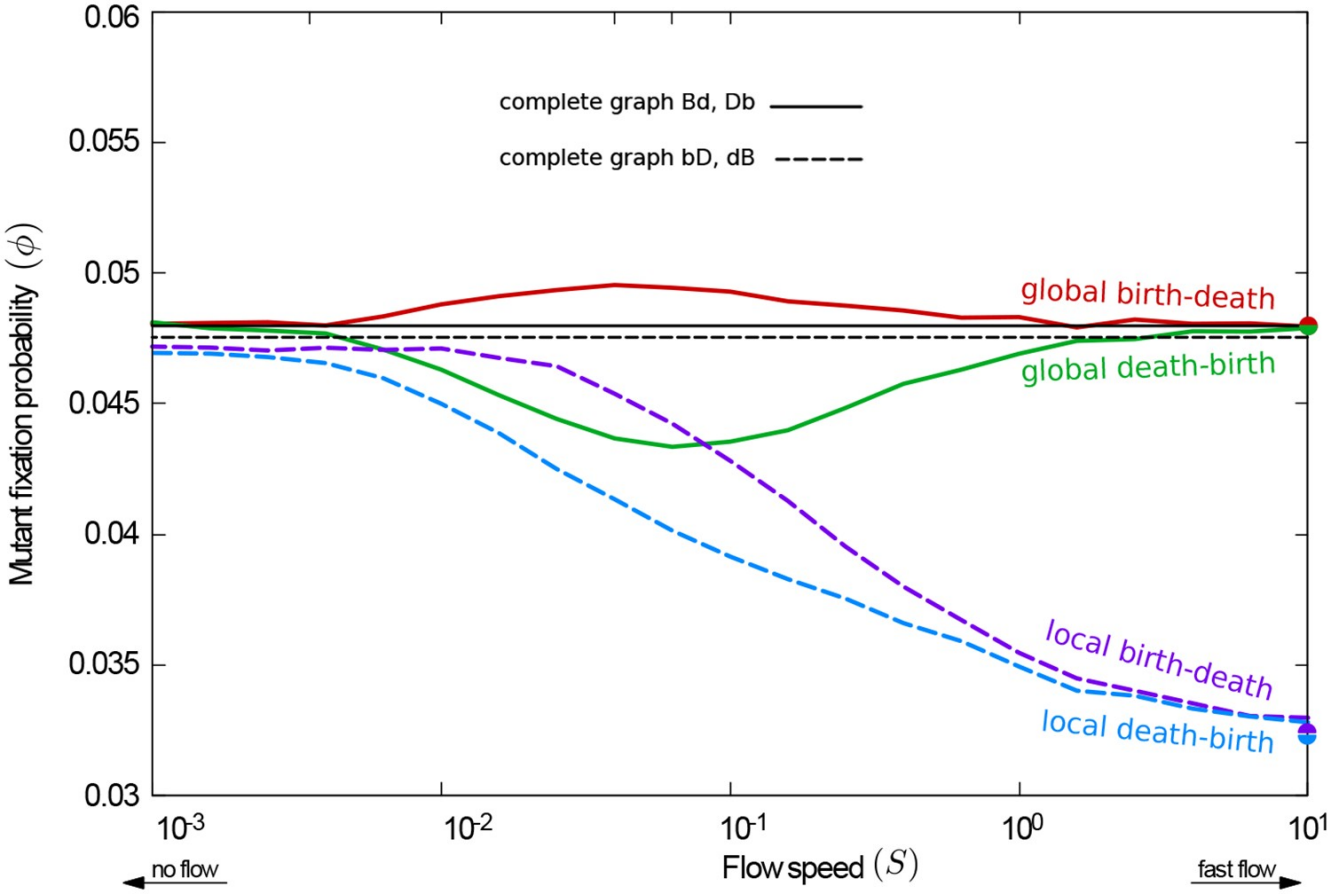
Fixation probability at different flow speeds for simulations started from a square lattice. For the global death-birth process a minimum of fixation probability is found at intermediate flow speeds; conversely, the global birth-death process shows a maximum. For the local processes no extrema are found; instead varying the flow speed interpolates monotonously between the behaviour on fixed lattices and the limit of fast flows.

In order to relate the success of mutants in populations advected by flows to these benchmark results, we show the outcome of simulations in which individuals are initially placed on the nodes of a regular lattice in Fig 7. Broadly, three different regimes can be distinguished:

#### Quasi-isothermal regime

On the left-hand side of Fig 7 (slow flows) fixation probabilities for all processes are approximately the same as on complete graphs. This is to be expected; in the limit of slow flows the evolutionary process concludes before the lattice structure is modified. The interaction network remains regular and, in line with the isothermal theorem, the fixation probability for global processes (continuous lines) is the one known from complete graphs; results for local processes (dashed lines) only differ slightly from *ϕ*_*CG*_.

With the periodic parallel shear flow, this agreement extends to slow, but non-vanishing flows. As described in the Methods section, during the first half of each period the flow moves the particles only vertically, with velocities dependent on their horizontal position. This means that some elements of the initial lattice remain intact; for example initial ‘columns’ of individuals (those with the same horizontal coordinate) move jointly. There is then only limited variation in the degree of the nodes in the network, and the interaction graph remains nearly regular. If fixation or extinction occurs before the flow disrupts this quasi-isothermal structure, the predictions of the isothermal theorem remain a good approximation. The flow speed above which this is no longer the case can be estimated from a comparison of the the time until the lattice structure is disrupted and the time-to-fixation; see part A of S1 Text in the Supporting Information for further details.

#### Intermediate regime

At intermediate flow speeds the fixation probability for the **Bd** process exhibits a maximum; a minimum is found for the **Db** process. These features can be related to the amplification or suppression effects on heterogeneous graphs, discussed in the previous sections. For intermediate flow speeds, the individuals’ motion is fast enough to distort the initial lattice structure before the evolutionary process concludes. On the other hand the flow is also sufficiently slow so that evolution has time to organise in clusters on the heterogeneous interaction network. Effectively evolution takes place on a slowly moving heterogeneous graph. This heterogeneity, in conjunction with the clustering of species, leads to amplification of selection for birth-death processes and suppression for death-birth processes. When selection is local this merely accelerates or delays the approach to the behaviour on complete graphs. When selection is global, however, the minimum (for **Db**) and maximum (for **Bd**) are generated. A rough estimate for the flow speed at which the extrema are seen can be obtained by comparing the time-to-fixation of the mutant species with the network renewal time; details can be found in part A of S1 Text in the Supporting Information.

#### Fast flow

In this regime the positions of individuals in space at each evolutionary event are essentially random, and the set of neighbours of any one particle is uncorrelated from an evolutionary event to the next one. The population is then ‘well-stirred’, and the analytical predictions from Ref. [79] apply.

## Discussion

We studied evolutionary dynamics in populations immersed in flows. In computer simulations, we measured the effect that the speed of the motion has on the success of an invading mutant, and found that the outcome of evolution can be affected by seemingly minor details of the model used to describe evolution.

Our results highlight the importance of including motion in the modelling of evolutionary dynamics. Just as static population structure can generate amplification or suppression of selection, we find that flow can act against or in favour of mutant invasion. While the models we study are stylised, we can identify general emerging principles. For instance, for the majority of evolutionary processes we observe a decrease in fixation probability when populations are in motion. This observation could be useful, for example, in industries where mutations are detrimental for the desired product but beneficial to the mutant, such as in microalgae, bacteria, fungi and yeast, relevant for the production of biodiesel [94–97]. Another example are the features we found to dominate fixation probability in the limits of very slow or very fast flows. If populations are mostly static in an experiment, our results indicate that whether selection acts locally or globally is a more important factor than the order of birth and death events. This is an important consideration for the choice of model to describe a particular system. If an experiment involves populations in motion, on the other hand, it is more important to decide whether to use a birth-death or a death-birth process as a model; in what step of evolutionary events competition takes place are less relevant in such situations. We note that the distinction between birth-death and death-birth processes requires an individual-based modelling approach; for example, no particular order of death and birth events is usually specified in models using continuous reaction-diffusion-advection equations.

It is appropriate to briefly comment on the limitations of our study. We focused on the periodic parallel shear flow in our simulations. However, we note that most features of the amplification or suppression of selection arise from the mixing of the population (i.e., the renewal of the set of neighbours of any one individual), and the heterogeneity of the interaction network. Both of these features can be expected in most real flows. While there may be quantitative differerences, we believe that the essence of our findings—modified selection strength with changing flow speed—is relevant beyond the exemplar of the shear flow. This is supported by observations in our earlier work [79], in which we obtained analytical results for the limit of fast flows, and demonstrated that these predictions are independent of many details of the flow field. At the same time, our work also opens up another view on the enhancement and suppression of selection due to mixing. We controlled the degree of mixing by changing the flow speed. That is to say, the neighbourhood of any one individual becomes increasingly less correlated in time as the flow speed increases. There might be other ways to generate such uncorrelated neighbourhoods. For example, it is known that certain flows enhance diffusive mixing [98]. One may therefore speculate that it might also be possible to obtain minimal or maximal fixation probability as the shape of the flow, and with it the degree of mixing, is varied at fixed flow speed.

Our study is also limited to frequency-independent selection; natural extensions would include more complex fitness functions to better model the experimental situation in ref. [53], where frequency-dependent fitness was identified for static conditions. While dilution techniques or resource-limited environments can be used to keep the population approximately constant in experiments without significantly modifying the mutants’ success [99], we note that future modelling work might relax the assumption of a fixed population size. This may be useful to explore the effects of demographic stochasticity. It is then also possible to introduce a carrying capacity. Such models can then account for localised regions of high densities, generated by the flow, and leading to areas in which the carrying capacity is exceeded, and birth is suppressed. This in turn can result in the collapse of that group of individuals, a potential effect not captured by our approach.

Despite the fact that direct measurements of the success of a specific mutation are not necessarily easy to perform, recent advances in technology make direct measurements of the fixation probability of a specific mutation feasible [23]. Experimental evidence of differences in fixation probabilities in static and in stirred populations can already be found in the literature [52–54]. In these studies, cultures of *E. coli* were grown in a continuously stirred liquid medium, on Petri dishes mixed every 24 hours, and on static Petri dishes. The structure and cluster formation of the cultures were found to have different dynamics under the different mixing conditions. The authors of ref. [54], for example, find that the ability to adapt, as measured by reproduction rates, is greater in the continuously-stirred case than in the case of only occasional mixing. This suggests a lower fixation probability in the slowly moving medium. Experimental observations of this kind highlight the relevance of studying the effects of motion on the mechanics of fixation. Our work provides an avenue to understanding the key factors affecting fixation in models of mobile populations.

## Supporting information

S1 Text Document containing additional text, simulations and figures. We further discuss the identification of relevant timescales for simulations started from regular lattices, the relevance of the number of active links, the effect of flow speed on dual selection processes, and the effects of temperature initialization.
(PDF)
