# Supplementary material for: Motion, fixation probability and the choice of an evolutionary process
Source: PLoS Comput Biol. 2019 Aug 5;15(8):e1007238. doi: 10.1371/journal.pcbi.1007238 (PMC6746388; doi:10.1371/journal.pcbi.1007238)
Supplement: S1 Text — We further discuss the identification of relevant timescales for simulations started from regular lattices, the relevance of the number of active links, the effect of flow speed on dual selection processes, and the effects of temperature initialization. (PDF) [file pcbi.1007238.s001.pdf]

Supporting Information

Motion, fixation probability and the choice of an evolutionary process

Francisco Herrerías-Azcué[1], Vicente Pérez-Muñuzuri[2] and Tobias Galla[1]
francisco.herreriasazcue@manchester.ac.uk, vicente.perez@cesga.es, tobias.galla@manchester.ac.uk

[1]Theoretical Physics, School of Physics and Astronomy,
The University of Manchester, Manchester M13 9PL, United Kingdom

[2]Group of Nonlinear Physics, Faculty of Physics,
University of Santiago de Compostela E-15782 Santiago de Compostela, Spain

A. IDENTIFICATION OF RELEVANT TIMESCALES FOR SIMULATIONS STARTED FROM REGULAR LATTICES

In the Results section of the main text we discuss the behaviour of the population when the initial condition is a regular lattice. We identify three different regimes: fast flows, intermediate flow speeds, and the quasi-isothermal regime. Here we discuss how these regimes can be identified from the simulations, and show how the time scales for the network renewal and quasi-isothermal regimes can be obtained. Simulations in this section were all initialized from a square lattice. The flow is the periodic parallel shear flow, with parameters as given in the main text.

A.1. End of quasi-isothermal regime

Fig. A.1 shows the average number of components in the interaction graph, the average size of each component, and the average degree in the network as a function of time.

We observe three different phases:

(i) At first, there is a very short interval ($t \lesssim 0.05$), in which there is only one component of size N. The lattice interaction graph is still intact.

(ii) Next, for $0.05 \lesssim t \lesssim 0.55$ we see an oscillating number of components; this corresponds to a period in which movement is only vertical (for the most part). Links between individuals with the same horizontal coordinate, i.e., within a 'column' of the original lattice, are not modified. The interaction graph is nearly regular.

(iii) Following this, we see a sharp decline of the average degree. The network cannot be considered isothermal any more, as fragmentation into heterogeneous separate components has begun. All measured quantities approach their stationary asymptotic values (the figure shows an average over multiple runs). Initial positions are washed out and the individuals take random positions in space.

We refer to the two initial stages (i) and (ii) as the '*quasi-isothermal*' period. If fixation (or extinction) is reached in most runs during this initial period, we expect fixation probabilities close to the one on the complete graph. For the model parameters in our simulations, phase (ii) ends at $t_{\mathrm{q}} \approx 0.55$, shortly after the first half period of the flow ($T/2 = 0.5$). This is marked with a dotted grey line in Fig. A.1. We note that the notion of the quasi-isothermal regime relies on the conservation of elements of the regular lattice; this may not be the case in other flows, for example if the motion of particles is not strictly vertical or horizontal. However, regardless of the type of flow, a critical flow speed can be found, below which the evolutionary process concludes before the lattice structure is significantly modified. Therefore, distinction of the regimes remains broadly valid.

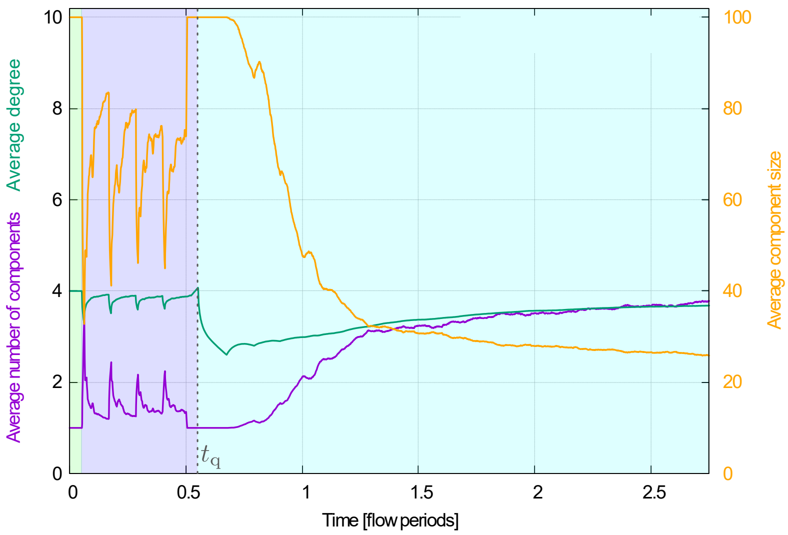

FIG. A.1. **Component formation as flow destroys initial lattice configuration.** The average number of components (purple) and the average degree (green) are plotted on the left axis; the average component size (orange) is plotted on the right axis. The three phases of the motion described in Sec. A.1 of the Supplementary Information are shaded in different colours. The grey dotted line at t_q marks the end of the phase in which the graph is quasi-isothermal.

A.2. Network renewal time

The time needed for neighbourhoods of individuals to lose correlation defines the *renewal* time, t_r. To measure this we have looked at the persistence of links in the network. As in ref.[1], we consider the probability that two nodes, connected at time t_0, are still connected at time $t_0 + t$; we write q_1 for this probability. Similarly we also measure the probability that two individuals who are not connected at t_0, are neighbours at time $t_0 + t$; we denote this probability by q_0. In the stationary state (i.e. for large t_0), the time, t, at which $q_0 \approx q_1$ is a good estimate for the time it takes for the network to be 'renewed'. Results are shown in Fig. A.2. For the parameters used throughout this paper, we estimate the renewal time as $t_r \approx 6.4$; this is the first time, t, for which both q_0 and q_1 are within 0.1% of their asymptotic value, q. Recalling that the flow period is $T = 1$, this indicates that the set of neighbours of any one individual in the population is uncorrelated from the set of neighbours of the same individual approximately six and half periods earlier.

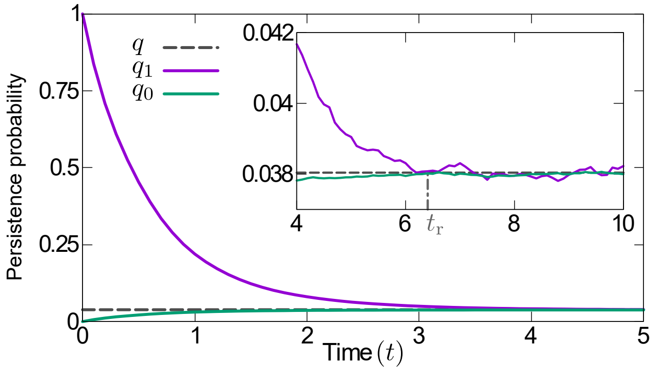

FIG. A.2. **Network-renewal time measured from the persistence of links.** Continuous lines show the probability that two nodes, connected (purple) or disconnected (green) at t_0 are still connected/disconnected at time $t_0 + t$. The dashed grey line shows the asymptotic value; the time needed for both probabilities (q_1 and q_0) to reach this value is the time it takes to renew the network. Both quantities are within 0.1% of their asymptotic value for the first time at $t_r \approx 6.4$, marked by a vertical dash-dotted grey line in the inset.

A.3. Conversion into characteristic flow speeds

The times t_q and t_r can be used to obtain estimates of the characteristic flow speeds separating the different regimes of dynamics described in the main text. These estimates are obtained by comparing t_q and t_r to the time-to-fixation for different flow speeds, S.

This is shown in Fig. A.3, where we plot the the time, t_1, required for a single mutant to reach fixation. Data is shown as a function of S. The network renewal time t_r is marked with a dash-dotted line on the vertical axis of the main panel, and t_q is marked with a dotted line on the vertical axis of the inset. The flow speeds for which $t_1 = t_q$ and $t_1 = t_r$ define flow speeds S_q and S_r, respectively. Due to differences in the mean fixation times, we note that the estimates for S_q and S_r vary between the different evolutionary update rules.

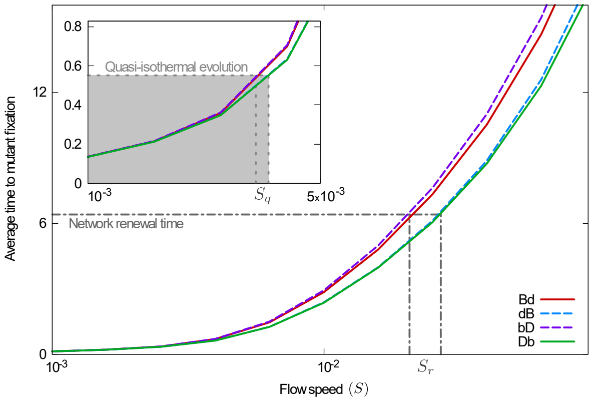

FIG. A.3. **Identification of time scales and flow speeds for the different evolutionary regimes.** The time to mutant fixation is plotted for the different evolutionary processes as a function of S. The flow speed at which the quasi-isothermal regime ends (S_q) is identified as the speed at which the mean time to fixation coincides with the time needed to significantly disrupt the interaction network, obtained in Fig. A.1. Similarly, S_r is the flow speed at which the mean fixation time agrees with the network renewal time, obtained in Fig. A.2.

These flow speeds are shown in the context of the fixation probability at different flow speeds in Fig. A.4. As expected, the S_q (grey dotted lines) marks the end of the quasi-isothermal regime. For flow speeds $S < S_q$ the mean time to fixation is shorter than the time t_q it takes the flow to significantly disrupt the initial lattice. This is the quasi-isothermal regime. For $S > S_q$ fixation is usually reached when the lattice has been significantly distorted.

The grey dash-dotted lines in Fig. A.4 correspond to S_r. The location of the extrema of the fixation probability for **Bd** and **Db** processes are found at flow speeds of the same order of magnitude as S_r. Similar observations were made for the time-to-consensus in a voter model in[2].

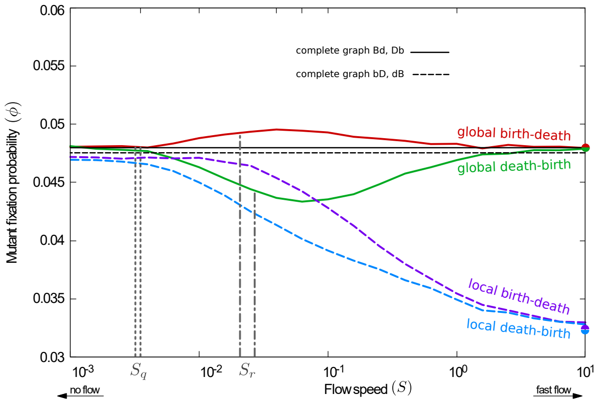

FIG. A.4. **Identification of time scales and flow speeds for the different evolutionary regimes.** Fixation probability at different flow speeds for simulations started from a square lattice are shown. S_q roughly corresponds to the speed marking the end of the quasi-isothermal regime; S_r is found to be of the same order of magnitude as the speed at the extrema of fixation probability.

B. RELEVANCE OF THE NUMBER OF ACTIVE LINKS

The amplification or suppression of selection, observed when comparing the simulations initialized from connected and unrestricted graphs, can be understood using an argument analogous to the one in ref.[3]. The number of active links does not change the probability with which a node is picked in the initial step of an evolutionary event, but it does have an effect on the choice of the second individual.

Wheel graphs are convenient to illustrate this. They consist of a central node (hub), connected to $N-1$ nodes organized in a circle around it (leaves), where N is the total size of the graph. The leaves are only connected to the hub and to their nearest neighbours on the rim of the wheel (see Fig. B.1 A). If two mutants are placed on a graph of this type, their location affects the number of active links (L_{act}), even though the total population size, the number of mutants and the degree of either mutants or wildtypes in the population remain unchanged. This is shown in Fig. B.1 B and C.

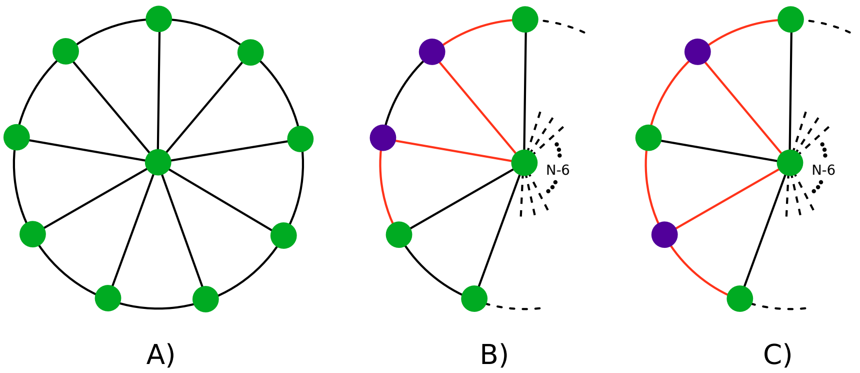

FIG. B.1. **Wheel graphs.** A) A sample wheel graph of size 10; B) A portion of a wheel graph with two mutants on adjacent leaves, with $L_{\text{act}} = 4$; C) A portion of a wheel graph with two mutants on non-adjacent leaves, with $L_{\text{act}} = 6$. Active links are marked orange. For B and C, $N-6$ wildtype nodes are not shown.

When the two mutants are neighbours (B), the network has 4 active links. If they are not neighbours (C), $L_{\text{act}} = 6$. To be able to have both settings one needs $N \geq 5$. In the two cases, both mutants have degree 3, and there are $N-3$ wildtypes with degree 3, and one with degree $N-1$. The probability that a mutant is replaced by a wildtype, however, is not the same for the two cases.

To see this, we focus on the case of neutral selection. We write $p_{m,w}$ for the probability that, in a single evolutionary event, a mutant is chosen first and a wildtype second. Similarly $p_{w,m}$ is the probability that a wildtype is chosen first and a mutant second. We then look at the ratio $Q = p_{m,w}/p_{w,m}$, to determine which one of these is more likely.

The probability that the individual picked in the first step is a mutant is $p_m = 2/N$; for a wildtype, $p_w = (N-2)/N$. This is the case in both configurations, B and C in Fig. B.1. For configuration B we then have:

$$p^B_{m,w} = \frac{2}{N}\left[\frac{1}{2}\left(\frac{2}{3}+\frac{2}{3}\right)\right] = \frac{4}{3N},$$
$$p^B_{w,m} = \frac{N-2}{N}\left[\frac{1}{N-2}\left(\frac{2}{N-1}+2\frac{1}{3}\right)\right] = \frac{2}{N}\frac{N+2}{3(N-1)}.$$

From this we find

$$Q_B = \frac{\frac{4}{3N}}{\frac{2}{N}\frac{N+2}{3(N-1)}} = \frac{2(N-1)}{N+2} \tag{B.1}$$

For configuration C we find

$$p^C_{m,w} = \frac{2}{N}\left[\frac{1}{2}\left(\frac{3}{3}+\frac{3}{3}\right)\right] = \frac{2}{N},$$
$$p^C_{w,m} = \frac{N-2}{N}\left[\frac{1}{N-2}\left(\frac{2}{N-1}+4\frac{1}{3}\right)\right] = \frac{2}{N}\frac{2N+1}{3(N-1)},$$

and hence

$$Q_C = \frac{\frac{2}{N}}{\frac{2}{N}\frac{2N+1}{3(N-1)}} = \frac{3(N-1)}{2N+1} \quad \text{(B.2)}$$

Comparing Eqs. (B.1) and (B.2), and assuming $N \geq 5$, we find $Q_B > Q_C$, i.e., the scenario with more active links (C) is more likely to result in events in which a wildtype is picked first and a mutant second. For a death-birth process, this means that mutants are more likely to reproduce, and for a birth-death process that they are more likely to die. This is in line with the results obtained in Fig. 5 of the main text.

A further configuration on a the wheel graphs is possible, placing one of the two mutants in the hub, which results in $L_{\text{act}} = N$. A similar analysis leads to $Q = (5N-8)/[N(N-1)]$, which is even smaller than Q_C. We note however that the average degree of mutants and wildtypes in this scenario is different from those in the other two settings.

C. DUAL SELECTION PROCESSES

As mentioned in the Methods section of the main text, the most general process of the birth-death or death-birth type is one that includes selection in both steps. In line with ref.[1], we call these 'dual-selection' processes; we label them **BD** and **DB**, respectively.

In this section, we present and discuss the simulation results for fixation probabilities at different flow speeds for both dual-selection processes. These are shown in Fig. C.1 for all configurations of initial positions studied in the main text: RGGs, CRGGs, and the square lattice.

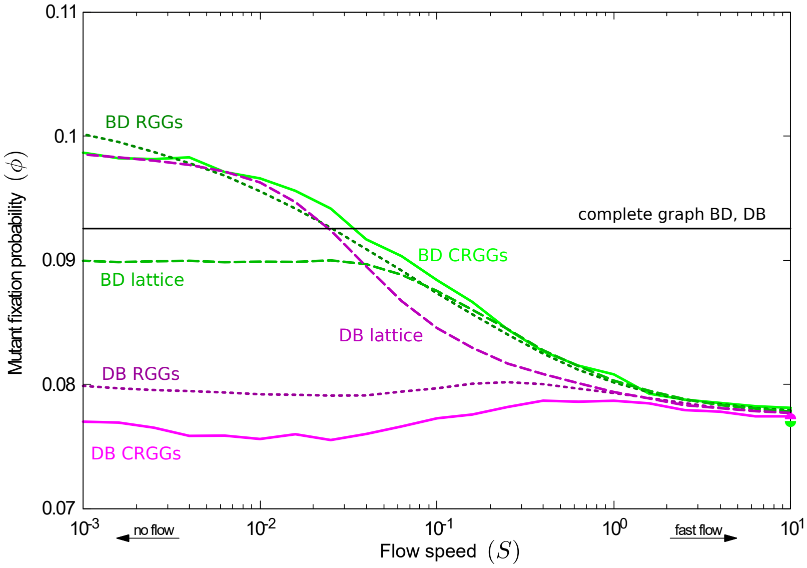

FIG. C.1. **Fixation probability at different flow speeds for dual-selection processes.** The result for the complete graph is plotted as a reference. Continuous lines correspond to simulations initialized from CRGGs, dotted lines to unrestricted RGGs, and dashed lines to simulations started from a lattice.

Naively, one could expect to be able to describe the response of the fixation probabilities to the flow speed in dual-selection processes through a combination of those observed for the corresponding global and local processes. That is, for **BD** one would expect to see similar features as in **Bd** and **bD**, and the behaviour of **DB** could be expected to show elements of that of **Db** and **dB**. For example, both **BD** and **DB** involve local selection, and for fast flows we find that the resulting fixation probability is lower than that of the complete graph, as is the case for both local processes, **bD** and **dB**.

For slow flows we make the following observations:

Dual-selection birth-death process: For simulations initialized on RGGs and CRGGs, the fixation probability of the **BD** process (dotted and continuous green lines in Fig. C.1) is above the one on complete graphs, as is the case for both **Bd** and **bD** (see red and purple lines in Fig. 5 of the main text).

If simulations are started from a lattice (dashed green line in Fig. C.1), the fixation probability of the **BD** process is slightly below ϕ_{CG}; this could also be expected, as the isothermal theorem does not hold due to the presence of local selection; we note that the fixation probability of the **bD** process is below that on a complete graph (see purple dashed line in Fig. 7 of the main text).

Interestingly, at slow flow speeds random initial positions act as an amplifier of selection (compared to the complete graph) for the **BD** process, whereas suppression of selection is observed for lattice initial positions.

Dual-selection death-birth process: The fixation probability of the **DB** process in simulations started on RGGs and CRGGs (dotted and continuous magenta lines in Fig. C.1) is well below ϕ_{CG}; we note that the mutant's success for both **Db** and **dB** is below that on a complete graph (see green and blue lines in Fig. 5 of the main text), so it is not surprising that the **DB** process shows this feature as well.

However, if simulations are started from a lattice (dashed magenta line in Fig. C.1), the fixation probability of the **DB** process is above ϕ_{CG}. This is different from both the **Db** or **dB** processes, who both lead to $\phi \leq \phi_{CG}$ (see green and blue lines in Fig. 7 of the main text). This indicates that simple intuition may fail – features present in **Db** and in **dB** processes may be altered when selection acts in both the death and the birth step. This, we believe is an unexpected observation, which could be pursued in future work. In particular it would be interesting to test when exactly such counter-intuitive behaviour is found when combining local and global selection, i.e., for example, for what types of graphs does this occur, and what common features do these graphs have?

We also note that, in contrast to the **BD** process, we find amplification of selection for the **DB** process when starting from regular lattice and slow flow. For random initial positions we find suppressed selection.

D. TEMPERATURE INITIALIZATION

The main text focuses on uniform initialization, that is, scenarios in which the initial mutant is chosen uniformly at random among the whole population. For example, this could describe situations, in which the mutation arises external to the population and where the mutant enters into the population due to transport from an external environment.

It is interesting, however, to consider the case in which the mutation arises during a reproduction event. The biologically most plausible scenario would then be captured by what is referred to as 'temperature initialization', see e.g. [4–7]. In this setting, the initial mutant is placed at a given node with a probability proportional to the so-called 'temperature' of the node. This temperature is given by the sum of the inverse degrees of the node's neighbours. High-temperature nodes are more 'active', in the sense that they take part in evolutionary events more often than nodes with lower temperatures. Therefore, if mutations arise in reproduction events they are more likely to occur at high-temperature nodes.

Compared to the case of a uniform placement of the mutant, temperature initialization leads an increased degree of the initial mutant; this shift of the mutant's degree is shown in Fig. D.1.

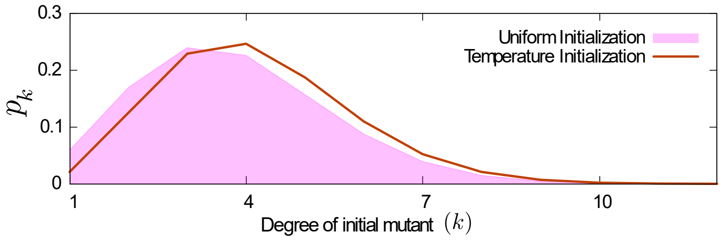

FIG. D.1. **Effect of initialization on the degree distribution.** The initial mutant's degree distribution is shown for uniform initialization (filled curve) and temperature initialization (line). The distribution is shifted to higher degrees when temperature initialization is used, which changes the overall fixation probability obtained in the limit of slow flows.

The effect that the shift of the mutant degree has on the fixation probability can be seen in Fig. D.2. The lines in the figure show the fixation probability of a single mutant for different flow speeds; simulations are started on CRGGs.

As expected, when the flows are sufficiently fast (right edge of Fig. D.2) the type of initialization has no effect. In this limit the interaction network undergoes sufficient mixing between evolutionary events, and therefore the distinction between high-temperature or low-temperature nodes is not meaningful.

In the case of slow flows the degree distribution of the initial mutant is a main factor contributing to the mutant's fixation probability (see also Figs. 3 and 4 in the main text). Temperature initialization hence results in a different fixation probability for slow flows; the limiting values for the no-flow case are shown in Fig. D.2 as triangular markers.

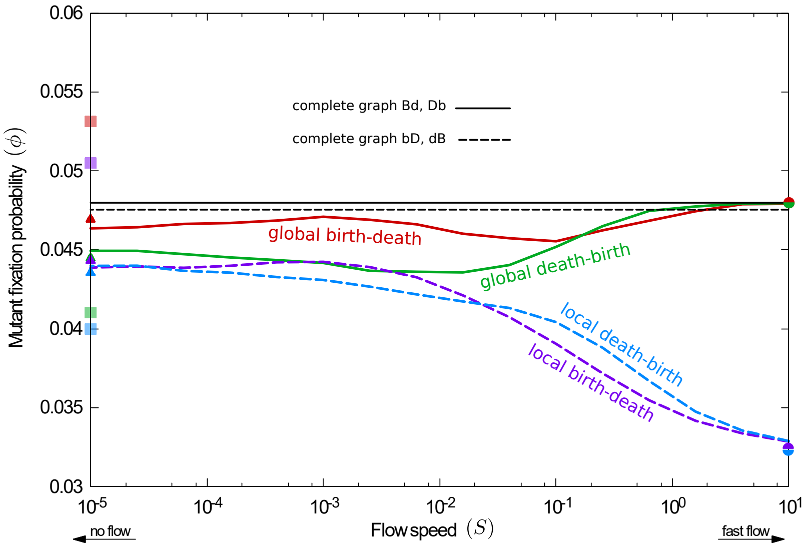

FIG. D.2. **Fixation probability at different flow speeds for temperature initialization.** The result for the complete graph is plotted as a reference. Simulations are initialized on CRGGs. Triangular markers show the value of ϕ obtained in the limit of slow flows, using the degree distribution in Fig. D.1. Square markers correspond to uniform initialization, and are shown only for comparison. The fast flow limit is unaffected by initialization.

Square markers in the figure show the limiting result for uniform initialisation, and as can be seen in the figure the fixation probabilities in the no-flow limit are affected considerably by the type of initialisation.

At intermediate flow speeds we observe features similar to those seen for uniform initialisation. For example, a minimum is found for the **Db** process (green line in Fig. D.2) at approximately $S \approx 10^{-2}$, and maxima are found for **Bd** and **bD** (red and purple, respectively) at around $S \approx 10^{-3}$. Similar effects are observed in Fig. 5 of the main text, discussed in the same section. However, we also observe a new characteristic in the response of the **Bd** process to the flow. A minimum can be seen at $S \approx 10^{-1}$, which indicates suppression of selection around these flow speeds. Interestingly, this coincides with the location of the maxima obtained for uniform initialization on the lattice, as seen in Fig. 7 of the main text, or Fig. A.4. We note that lattices are isothermal graphs, temperature and uniform initialization are hence identical. As discussed in Sec. A of this Supplement, these speeds roughly correspond to situations in which the fixation time is similar to the renewal time of the network. It is therefore to be expected that the initial placement of the mutant affects its fixation probability.

Intuition regarding the observed suppression of selection can be obtained with the same arguments presented in the main text. Namely, highly connected nodes are more likely to die under birth-death dynamics. Since temperature initialization effectively samples from highly connected nodes (relative to the rest of the population), the fixation probability of the initial mutant is decreased.

Suppression of selection at $S \approx 10^{-1}$ is not discernible in Fig. 5 for two reasons. First, uniform initialization does not favour highly connected nodes for the initial mutant, and so there are no effects due to high connectivity of the mutant. Second, the fixation probability in the slow-flow limit for uniform initialization is above that of the fast flow limit; therefore the interpolation between the two limits naturally produces a negative slope at flow speeds $S \approx 10^{-1}$.

Although temperature initialization seems to modify the response of the fixation probability of the initial mutant to the speed of the flow, the principles behind the amplification or suppression of selection are the same as the ones discussed in the main text, i.e. the connectedness of the initial mutant (which allows us to compare the slow flow with fixed graphs), the formation of clusters (which results in the extrema at $S \approx 10^{-2} - 10^{-3}$), and the loss of correlation (in time) of the set of neighbours of a given individual for fast flows.

[1] Francisco Herrerías-Azcué, Vicente Pérez-Muñuzuri, and Tobias Galla, "Stirring does not make populations well mixed," Scientific Reports **8**, 4068 (2018).
[2] Tobias Galla and Vicente Pérez-Muñuzuri, "Time scales and species coexistence in chaotic flows," EPL (Europhysics Letters) **117**, 68001 (2017).

[3] Tibor Antal, Sidney Redner, and Vishal Sood, "Evolutionary Dynamics on Degree-Heterogeneous Graphs," Physical Review Letters **96**, 188104 (2006).

[4] Erez Lieberman, Christoph Hauert, and Martin A. Nowak, "Evolutionary dynamics on graphs," Nature **433**, 312–316 (2005).

[5] Mark Broom, Jan Rychtář, and B. T. Stadler, "Evolutionary Dynamics on Graphs - the Effect of Graph Structure and Initial Placement on Mutant Spread," Journal of Statistical Theory and Practice **5**, 369–381 (2011).

[6] Ben Adlam, Krishnendu Chatterjee, and Martin A. Nowak, "Amplifiers of selection," Proceedings of the Royal Society A: Mathematical, Physical and Engineering Science **471**, 20150114 (2015).

[7] Andreas Pavlogiannis, Josef Tkadlec, Krishnendu Chatterjee, and Martin A. Nowak, "Construction of arbitrarily strong amplifiers of natural selection using evolutionary graph theory," Communications Biology **1**, 71 (2018).
